# A Case Report of Subacute Thyroiditis following mRNA COVID-19 Vaccine

**DOI:** 10.1155/2021/8952048

**Published:** 2021-11-11

**Authors:** Leidy Plaza-Enriquez, P. Khatiwada, M. Sanchez-Valenzuela, A. Sikha

**Affiliations:** ^1^Department of Internal Medicine, Memorial Healthcare System, Hollywood, FL, USA; ^2^Internal Medicine Residency, St. Barnabas Hospital, Bronx, NY, USA

## Abstract

**Background:**

Subacute thyroiditis has been reported after administration of influenza vaccine. We describe a case of a patient who developed subacute thyroiditis after administration of. Moderna mRNA COVID-19 vaccine. *Case Presentation*. A 42-year-old female, with a past medical history of stage IIIB pT3N1aM0 right adenocarcinoma of colon status, after right hemicolectomy on 01/2020, followed by adjuvant chemotherapy, paroxysmal supraventricular tachycardia, iron deficiency anemia, chemotherapy-induced neuropathy, and lumbar radiculopathy, presented to our clinic with anterior neck pain that started 6 days after the second dose of Moderna mRNA COVID-19 vaccine. She was diagnosed with subacute thyroiditis and treated conservatively with pain medications.

**Conclusion:**

Subacute thyroiditis could represent one of the side effects of Moderna mRNA COVID-19 vaccine. Further reports are lacking.

## 1. Introduction

Subacute thyroiditis is a well-known clinical diagnosis, usually presenting one to two weeks after an acute viral infection, including COVID-19 [1,2]. Symptoms fluctuate in intensity and last for 3 to 6 weeks. Laboratory investigations show high erythrocyte sedimentation rate (ESR), elevated C-reactive protein (CRP), and suppressed TSH with elevated free T4 and T3 [2]. Treatment with analgesics is recommended, and prednisone if poor improvement [2,3].

Subacute thyroiditis has been reported after administration of influenza and COVID-19 vaccines [1,4–6]. We present a case of subacute thyroiditis after administration of an mRNA COVID-19 vaccine. To our knowledge, this is the first case report of subacute thyroiditis after Moderna mRNA COVID-19 vaccine.

## 2. Case Report

A 42-year-old African American female with a past medical history of stage IIIB pT3N1aM0 right adenocarcinoma of colon status after right hemicolectomy on 01/2020, followed by adjuvant chemotherapy, paroxysmal supraventricular tachycardia, iron deficiency anemia, chemotherapy-induced neuropathy, and lumbar radiculopathy, presented to our primary-care clinic on the first week of March 2021 complaining of bilateral ear pain for 2 weeks. The patient was treated with 9 cycles of FOLFOX regimen (fluorouracil, oxaliplatin, and leucovorin) for her colon carcinoma and completed her chemotherapy on 09/2020. She received her 1^st^ dose of Moderna mRNA COVID-19 vaccine on 02/05/2021 and the second dose on 03/05/2021. Symptoms started about 5–6 days after the vaccination. She began experiencing right-sided and then left-sided earache radiating down to the lateral and anterior neck and bilateral lower jaw. The pain was exacerbated by turning her head, swallowing, and coughing. She denied fever, fatigue, malaise, anorexia, myalgia, dysphagia, dysphonia, dyspnea cough, rhinorrhea, lacrimation, and ear discharge. She has no history of thyroid disease and/or head and neck radiation exposure. The patient denied any family history of head and neck cancer. She denied smoking cigarettes, but drinks alcohol socially. Her home medications included Metoprolol 25 mg two times a day, vitamin B12 1000 mcg once daily, ferrous sulfate 325 mg two times a day, and multivitamin capsule 1 capsule daily.

Her vitals at the time of presentation were a temperature of 35.9°C, pulse 88 beats/min, blood pressure 111/75 mm of Hg, respiratory rate 17 breaths/min, and oxygen saturation 97% on room air. Physical examination was notable for anterior neck swelling about 3 cm in size which was tender on palpation.

**Figure 1:**
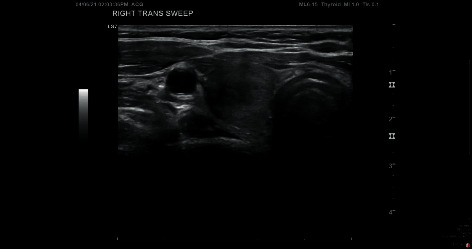
Ultrasound of the right thyroid lobe showing a heterogeneous mass.

**Figure 2:**
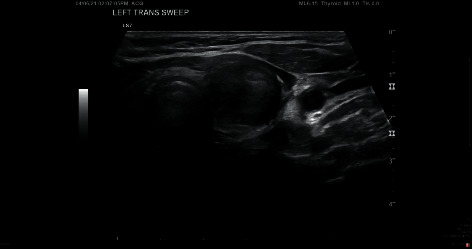
Ultrasound of the left thyroid lobe showing a heterogeneous mass.

Routine laboratory investigations showed WBC 6.4 (3.5–10.0 1000/uL), hemoglobin 13.2 g/dL (1.4–15.4 g/dL), platelets 207 (150–450 1000/uL), BUN 10 mg/dL (7–18 mg/dL) creatinine 0.43 (0.51–0.95 mg/dL), and normal electrolyte level and liver function test. Laboratory investigations were significant for low serum TSH < 0.0005 IU/ml (0.350–4.000 IU/ml), low serum T3 1.90 ng/dL (0.60–1.81 ng/mL), and normal free T4-1.51 ng/dL (0.80–2.00 ng/dL). TSH the week prior to COVID-19 vaccination, obtained as a part of fatigue workup, was 2.030 IU/ml (0.350–4.000 IU/ml). ESR was elevated 81 mm/hour. Thyroid peroxidase antibodies were negative <10 (<35 IU/ml). Ultrasound of the thyroid revealed a 1.6 × 1.4 × 3.4 cm heterogeneous mass in the right lobe and a 1.6 × 1.8 × 3.4 cm hypoechoic and moderately heterogeneous mass in the left lobe (Figures 1 and 2) without calcifications, associated with mildly enlarged inferior left paratracheal lymph nodes.

The patient was treated with analgesics as needed for pain, until she underwent ultrasound-guided fine-needle aspiration biopsy of the bilateral 3.4 cm thyroid masses in April 2021. Biopsy results showed scattered multinucleated giant cells (some with ingested colloid), scattered epithelioid granulomas, groups of follicular cells, scattered lymphocytes, and karyorrhexis. These features were consistent with a benign hyperplastic nodule suggestive of subacute (de Quervain) thyroiditis. Cytopathology was benign (Bethesda Category II). During this time period, she had minimal symptoms and was treated with NSAIDs as needed for pain. On a subsequent follow-up visit, 2 months after the biopsy, previously described anterior neck discomfort and earache radiating to the neck resolved. Her follow-up labs obtained on 06/2021 showed TSH 8.80 (0.350–4.000 IU/ml), free T4 0.65 ng/dL (0.80–2.00 ng/dL), total T3 1.20 ng/ml (0.60–1.81 ng/ml), and ESR 14 mm/hour.

## 3. Discussion

Subacute thyroiditis is a relatively uncommon cause of hyperthyroidism that has been associated with upper respiratory viruses, such as influenza, mumps, and other respiratory viruses, but few cases have been reported after inactivated viral vaccines or live-attenuated vaccines such as those for influenza [1,4,5]. This condition is characterized by neck pain, a tender goiter, and a thyroid dysfunction. We present a case of a female with classic symptoms of subacute thyroiditis after Moderna mRNA COVID-19 vaccine administration. Thyroiditis has been reported as a possible side effect of COVID-19 vaccine in 4 cases thus far [3,5], one case after CoronaVac® administration [5] and the other with Pfizer mRNA COVID-19 vaccine [3]. Cross recognition between the coronavirus spike protein targeted with mRNA vaccine and thyroid cell antigens is likely the mechanism. Differential diagnosis includes Graves' disease, toxic multinodular goiter, and pharyngitis, but after the administration of a vaccine, subacute thyroiditis should be suspected. Further reports are lacking; however, clinicians should consider subacute thyroiditis after administration of mRNA COVID-19 vaccines as a possible side effect.

## Abbreviations

ESR: Erythrocyte sedimentation rate
CRP: C-reactive protein
TSH: Thyroid-stimulating hormone
T3: Triiodothyronine
T4: Thyroxine.
